# Cardiovascular and renal outcomes with sodium glucose co-transporter 2 inhibitors in patients with type 2 diabetes mellitus: A system review and network meta-analysis

**DOI:** 10.3389/fphar.2022.986186

**Published:** 2022-11-24

**Authors:** Lei Tian, Sinan Ai, Huijuan zheng, Hanwen Yang, Mengqi Zhou, Jingyi Tang, Weijing Liu, Wenjing Zhao, Yaoxian Wang

**Affiliations:** ^1^ Department of Nephrology, Beijing Hospital of Traditional Chinese Medicine, Capital Medical University, Beijing, China; ^2^ Renal Research Institution of Beijing University of Chinese Medicine, Key Laboratory of Chinese Internal Medicine of Ministry of Education and Beijing, Dongzhimen Hospital Affiliated to Beijing University of Chinese Medicine, Beijing, China; ^3^ China-Japan Friendship Hospital, Beijing, China

**Keywords:** sodium glucose co-transporter 2 (SGLT2) inhibitors, type 2 diabetes mellitus, network meta-analysis, cardiovascular outcomes, renal outcomes

## Abstract

Cardiovascular and renal impairment are the most common complications of type 2 diabetes mellitus (T2DM). As an emerging class of glucose-lowing agents sodium glucose co-transporter 2 (SGLT2), possesses beneficial effects on cardiovascular and renal outcomes in patients with T2DM. The aim of this study is to assess the efficacy of different SGLT2 inhibitors for cardiovascular and renal outcomes for patients with T2DM when compared with placebo. We performed a systematic search of PubMed, Embase, and the Cochrane library from inception through November 2021. Randomized clinical trials enrolling participants with T2DM were included, in which SGLT2 inhibitors were compared with each other or placebo. The primary outcomes including all-caused mortality, Cardiovascular outcomes (cardiovascular mortality, hospitalization for heart failure), and the renal composite outcomes (worsening persistent microalbuminuria or macroalbuminuria, new or worsening chronic kidney disease, doubling of serum creatinine, end-stage renal disease, renal transplant, or renal death). The data for the outcomes were pooled and recorded as Hazard rations (HRs) with 95% confidence intervals (CLs). Two researcher independently screened the trials and drawn the data. Ten trials enrolling 68,723 patients were included. Compared with placebo groups, Canagliflozin [HR, 0.85 (95%CI, 0.75–0.98)], ertugliflozin [HR, 0.93 (95%CI, 0.78–1.11)], and sotagliflozin [HR, 0.94 (95%CI, 0.79–1.12)] were associated with a reduction in all-cause mortality. Canagliflozin [HR, 0.84 (95%CI, 0.72–0.97)], dapagliflozin [HR, 0.88 (95%CI, 0.79–0.99)], empagliflozin [HR, 0.62 (95%CI, 0.49–0.78)], ertugliflozin [HR, 0.92 (95%CI, 0.77–1.10)], and sotagliflozin [HR, 0.88 (95%CI, 0.73–1.06)] were associated with a reduction in cardiovascular mortality; Canagliflozin [HR, 0.64 (95%CI, 0.53–0.77)], dapagliflozin [HR, 0.71 (95%CI, 0.63–0.81)], empagliflozin [HR, 0.65 (95%CI, 0.50–0.85)], ertugliflozin [HR, 0.70 (95%CI, 0.54–0.90)], and sotagliflozin [HR, 0.66 (95%CI, 0.56–0.77)] were associated with a reduction in hospitalization for heart failure. Dapagliflozin [HR, 0.55 (95%CI, 0.47–0.63)], Empagliflozin [HR, 0.54 (95%CI, 0.39–0.74)], canagliflozin [HR, 0.64 (95%CI, 0.54–0.75)], sotagliflozin [HR, 0.71 (95%CI, 0.46–1.09)], and ertugliflozin [HR, 0.81 (95%CI, 0.63–1.04)] were associated with a reduction in the renal composite outcome. All SGLT2 inhibitors showed a reduction in cardiovascular mortality, hospitalization for heart failure, renal composite outcomes and all-cause mortality. Canagliflozin and empagliflozin seemed to have the same efficacy in reducing hospitalization for heart failure, but empagliflozin had advantage in reducing cardiovascular mortality, whereas dapagliflozin most likely showed the best renal composite outcomes.

## 1 Introduction

In 2021, the number of diabetic patients was estimated to be approximately 537 million, and by 2045, the prevalence is expected to reach 783 million (International Diabetes Foundation, 2021). The most frequent feature of diabetic patients is chronic or periodic hyperglycemia, which results in impaired organ function, such as macrovascular disease (including heart and brain disease), microvascular disease (including kidney and eye disease), as well as peripheral neuropathy (like paresthesia in hands and feet) (Bjornstad et al., 2021). Cardiovascular and renal impairment are the most common complications (Koye et al., 2018; Deng et al., 2021). Approximately half of the patients with type 2 diabetes mellitus (T2DM) develop diabetic kidney disease (DKD), which is the leading cause of end-stage renal disease (Anders et al., 2018; Umanath and Lewis, 2018). Additionally, the main cause of morbidity and mortality in patients with T2DM is cardiovascular complications (Lloyd-Jones et al., 2009; Fox et al., 2015). Thus, when choosing hypoglycemic drugs, lowering the blood glucose levels should be considered the primary therapeutic strategy as well as preventing cardiovascular and renal complications in patients with T2DM. Many antidiabetic medications are approved to treat patients with T2DM, such as metformin, insulin, and glitazones, but they have significant limitations for improving cardiovascular and renal function, and some may have side effects (Gilbert and Krum, 2015; Packer, 2018). Even the use of specific antidiabetic medications or intensive hypoglycemic may be related with adverse cardiovascular and renal vascular events antidiabetic medication**.** For example, the safety of sulfonylureas and insulin in heart failure is unclear. Thiazolidinediones (glitazones) have been showed to increased risk of cardiovascular events, so they should not be used in patients with heart failure or patients at high risk of heart failure (Gilbert and Krum, 2015; Seferović et al., 2018). A meta-analysis of thiazolidinediones further demonstrated that in patients with type 2 diabetes, rosiglitazone was associated with significantly higher odds of congestive heart failure, myocardial infarction, and death in real-world settings compared with pioglitazone (Loke et al., 2011).

As an emerging class of glucose-lowing agents, the pharmacodynamic mechanism of sodium glucose transporter 2 (SGLT2) inhibitors are to inhibit the glucose reabsorption in the proximal tubule and increase urinary glucose excretion to lower hyperglycemia (Nauck, 2014; Gallo et al., 2015). Additionally, SGLT2 inhibitors’ mechanism of action is insulin-independent and islet β-cell failure for antidiabetic therapies is not involved. The drug was also shown to be effective at all stages of T2DM for patients without renal impairment (Washburn, 2012; van Baar et al., 2018). The beneficial effects of SGLT2 inhibitors on patients with T2DM include the hypoglycemic effect as well as reducing body weight, increasing urinary sodium excretion, contracting intravascular volume, and changing renal hemodynamics (DeFronzo et al., 2017; Thomas and Cherney, 2018; Neuen et al., 2019). These effects might improve blood pressure, intrarenal blood flow, and albuminuria, and reduce the risk of cardiovascular and renal complications in patients with T2DM (DeFronzo et al., 2017; Thomas and Cherney, 2018). The Empagliflozin Cardiovascular Outcome Event Trial (EMPA-REG-OUTCOME) first reported that empagliflozin could reduce the risk of major adverse cardiovascular events (MACEs) including cardiovascular death, nonfatal stroke, and myocardial infarction, as well as prevent kidney disease end points, such as serum creatinine doubling, renal failure, and renal death (Zinman et al., 2015). Subsequently, the Canagliflozin Cardiovascular Assessment Study (CANVAS) program and the Dapagliflozin Effect on Cardiovascular Events-Thrombosis in Myocardial Infarction 58 (DECLARE-TIMI-58) trials, which were multi-center randomized controlled trials (RCTs), confirmed that these SGLT2s significantly protected against cardiovascular and renal events compared with placebo treatment in patients with T2DM (Neal et al., 2017; Wiviott et al., 2019). The result of the Canagliflozin and Renal Endpoints in Diabetes with Established Nephropathy Clinical Evaluation (CREDENCE) trial, which was a recent novel multicenter international clinical trial, indicated that canagliflozin remarkably reduced MACE and renal events in patients with T2DM and chronic kidney disease (Perkovic et al., 2019). As a gold standard phase III clinical trial, these results all confirmed the profitable effects of SGLT2 inhibitors on cardiovascular and renal outcomes in patients with T2DM. Some published systematic reviews and meta-analysis have summarized the effects of SGLT2 inhibitors on cardiovascular and renal outcomes in patients with T2DM, which suggests that SGLT2 inhibitors reduce the risk of cardiovascular and renal events with no additional safety concerns (Neuen et al., 2019; Toyama et al., 2019; Zelniker et al., 2019). On the basis of the encouraging results for SGLT2 inhibitors in preventing cardiovascular and renal events during clinical trials with patients with T2DM, the drug has been widely recommended among antidiabetic medications for patients with diabetes. SGLT2 inhibitors include canagliflozin, dapagliflozin, tofogliflozin, luseogliflozin, ipragliflozin, empagliflozin, ertugliflozin, and sotagliflozin (an inhibitor of SGLT2 and SGLT1). The following four SGLT2 inhibitors have been approved by the U.S. Food and Drug Administration (FDA) for the treatment of hyperglycemia as monotherapy or in combination with other glucose-lowering agents for patients with T2DM: canagliflozin, dapagliflozin, empagliflozin, and ertugliflozin (Tuttle et al., 2021). Several oral SGLT2 inhibitors are available to choose from, and determining the best SGLT2 inhibitor to prescribe for patients with T2DM who may have difference cardiovascular and renal risk can be difficult. Several previous network analyses with multiple categories of antidiabetic medications, including SGLT2 inhibitors, evaluated the efficacy of these drugs on cardiovascular and renal outcomes in patients with T2DM, but the conclusions were either uncertain or lacked relevance (Palmer et al., 2021).

Therefore, we performed a network meta-analysis to assess the benefits and limits of different of SGLT2 inhibitors for cardiovascular and renal outcomes in patients with T2DM. The aim of our analysis was to offer evidence for the clinical application of SGLT2 inhibitors in patients with T2DM.

## 2 Methods

### 2.1 Data sources and searches

This systematic review article was conducted in accordance with the Preferred Reporting Items for Systematic Reviews and Meta-Analyses (PRISMA-NMA). The network meta-analysis systematic review protocol was registered in the PROSPERO database (International Prospective Register of Systematic Reviews, https://www.crd.york.ac.uk/prospero, registration number CRD42020202202). A systematic search of PubMed, Embase, and the Cochrane library was performed from database inception to 11 November 2021 using the search terms “diabetes mellitus”, “type 2 diabetes”, “type II diabetes”, “sodium-glucose transporter two inhibitors”, “sodium glucose transporter two inhibitor”, “SGLT2 inhibitor”, “sodium glucose transporter II inhibitor”, “canagliflozin”, “dapagliflozin”, “empagliflozin”, “ertugliflozin”, “tofogliflozin”, “ipragliflozin”, and “remogliflozin”. The searches were limited to English-language articles. The selected documents were edited and managed using a bibliographic database created in EndNote X9.1(US) and duplicate documents were removed.

### 2.2 Study selection

We screened trials account of the following inclusion criteria. 1) They were randomized clinical trials (RCTs) that compared SGLT2 inhibitors (including dapagliflozin, canagliflozin, tofogliflozin, luseogliflozin, ipragliflozin, empagliflozin, ertugliflozin, or sotagliflozin) with placebo or other glucose-lowering treatments. 2) The participants were male or female individuals with T2DM who were ≥18 years old, and had HbA1c levels between 6.5% and 10.5%. 3) The duration of medication was ≥13 weeks 4) The outcomes of the trials included at least one cardiovascular or renal outcomes. The exclusion criteria were as follows. 1) The patients had type 1 diabetes mellitus or a history of hereditary glucose or galactose malabsorption. 2) The trials did not specify the inclusion or exclusion criteria. 3) Repeated use of data for secondary analyses. 4) Preclinical research of animal models.

### 2.3 Data extraction and quality assessment

Trial Selection and data extraction were conducted independently by two authors (LT and SA) based on the inclusion and exclusion criteria. The titles and abstracts of the trials were screened by two investigators (HZ and HY). Data were extracted using piloted forms in Microsoft Excel 2016 (US), and quality evaluation were conducted independently and in duplicate by two investigators (LT and SA). Disagreements were settled by deliberation with a third reviewer (WL and YW).

The outcomes in our network meta-analysis were mainly including the cardiovascular, renal outcome and all-cause mortality. Cardiovascular outcome included cardiovascular mortality, hospitalization for heart failure. Renal composite outcome was defined as a composite of doubling of serum creatinine level, initiation of renal-replacement therapy, or renal death.

The quality of the included studies and risk of trial bias were assessed using the Cochrane risk of bias assessment tool, which included randomization, quality of blinding, allocation concealment, and reporting bias categories. For each category, the trial was graded as high, low, or unclear. Two reviewers independently performed the data extraction and quality evaluation, and if there were any disagreements, they were resolved by discussion. The analyses were performed using Review Manager v5.1 (Cochrane Collaboration, http://www.cochrane.org).

### 2.4 Data synthesis and analysis

We used frequentist network meta-analysis. Hazard ratios (HRs) with 95% confidence intervals (CIs) were used to investigate the effects of cardiovascular and renal events. We used a frequentist approach to compare the effect of different SGLT2 inhibitor classes on these outcomes. The statistical package “netmeta” in R (version 4.1.2) was used for data processing. We used the forest function in “netmeta” package to plot the comparison forest, obtaining indirect comparison results and overall ranking by comparing to each intervention. Furthermore, the “netmeta” package can rank each intervention in order of merit by calculating the P-score of each intervention in the study. P-score from 0 to 1 was used to determine the probability of a treatment being the most effective (0 represents the worst; 1 represents the best). All the analyses results and plots were generated in R.

## 3 Results

**FIGURE 1:**
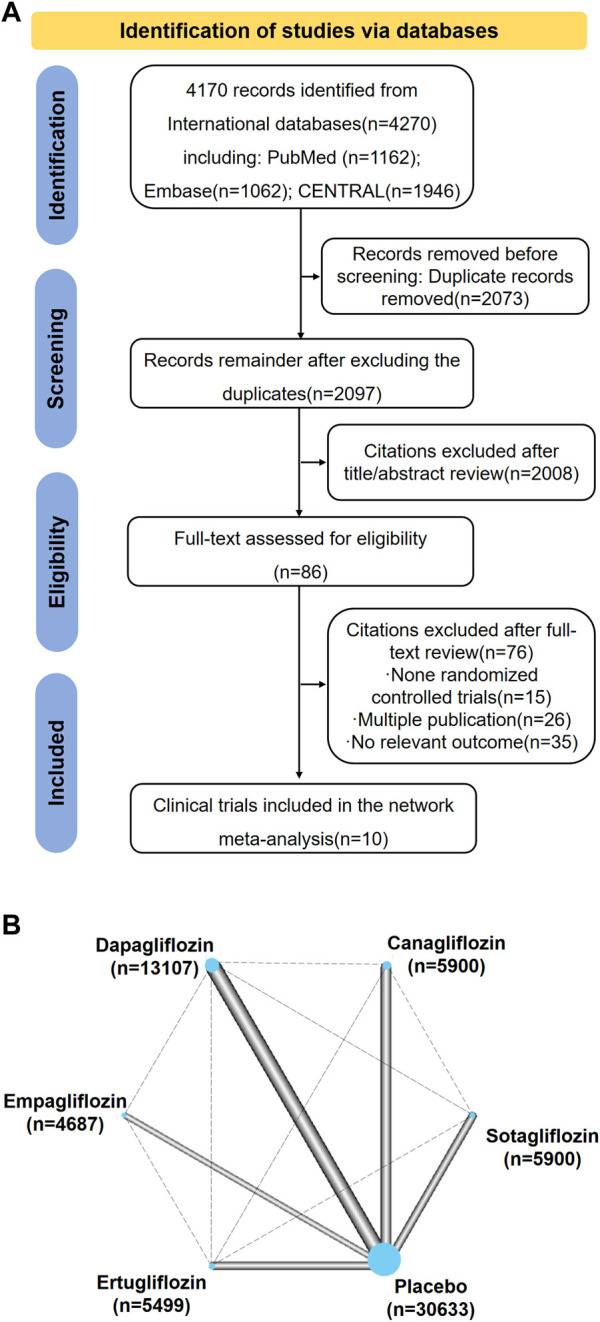
**(A)** Summary of Study Identification for Network Meta-analyses. **(B)** Network Plot for All Studies. Graphical representation of network for all induced trials. Connecting solid lines represent head-to-head comparisons between drugs, indicated by nodes. The thickness of lines between nodes is proportional to the number of trials comparing the treatments. The sizes of the nodes are proportional to the number of patients in each treatment which is marked below drugs. The control group is compared with each drug class. This is accounted for within the network model and does not constitute duplication of participants.

There were 4,170 records identified during the searches. Among them, 2,097 records remained after duplicates were excluded, but 2,008 additional records were excluded after reviewing their titles and abstracts, leaving 89 records for full-text review. Among them, ten trials were included for the network meta-analysis (Figures 1A,B). These trails consist of CANVAS, CANVAS-R, CREDENCE, DECLARE-TIMI58, DAPA-CKD, DAPA-HF, EMPA-REG, VERTIS-CV, SCORED, and SOLOIST-WHF (Zinman et al., 2015; Neal et al., 2017; McMurray et al., 2019; Perkovic et al., 2019; Wiviott et al., 2019; Cannon et al., 2020; Bhatt et al., 2021a; Bhatt et al., 2021b; Wheeler et al., 2021). The characteristics of the included trials are presented in Supplementary Table S1. There were 67,823 patients with T2DM who were randomly assigned to the SGLT2 inhibitor treatment or a comparator treatment (placebo or other SGLT2 inhibitor). Among the ten trials, the CREDENCE, SCORED, and DAPA-CKD trials enrolled patients who had both T2DM and kidney disease. The SOLOIST-WHF and DAPA-HF trials enrolled patients who had both T2DM and heart failure. All trials reported all-cause mortality. However, hospitalization for heart failure was not reported in DAPA-CKD, and renal composite outcomes were not reported in the SOLOIST-WHF and DAPA-HF trials. All trials compared a SGLT2 inhibitor with placebo.

### 3.1 Risk of bias and publication bias

The Cochrane Collaboration risk of bias assessment tool indicated that all of the included trials used randomized sequence generation, and the methods used and allocation concealment were clearly stated. All the trials included blinding for the participants and investigators. All included trials were categorized as having a low risk of detection bias, attrition bias, reporting bias, and other bias. All of the trials were assessed as having a low risk of publication bias.

### 3.2 Network meta-analyses of SGLT2 inhibitors on all-cause mortality

**FIGURE 2:**
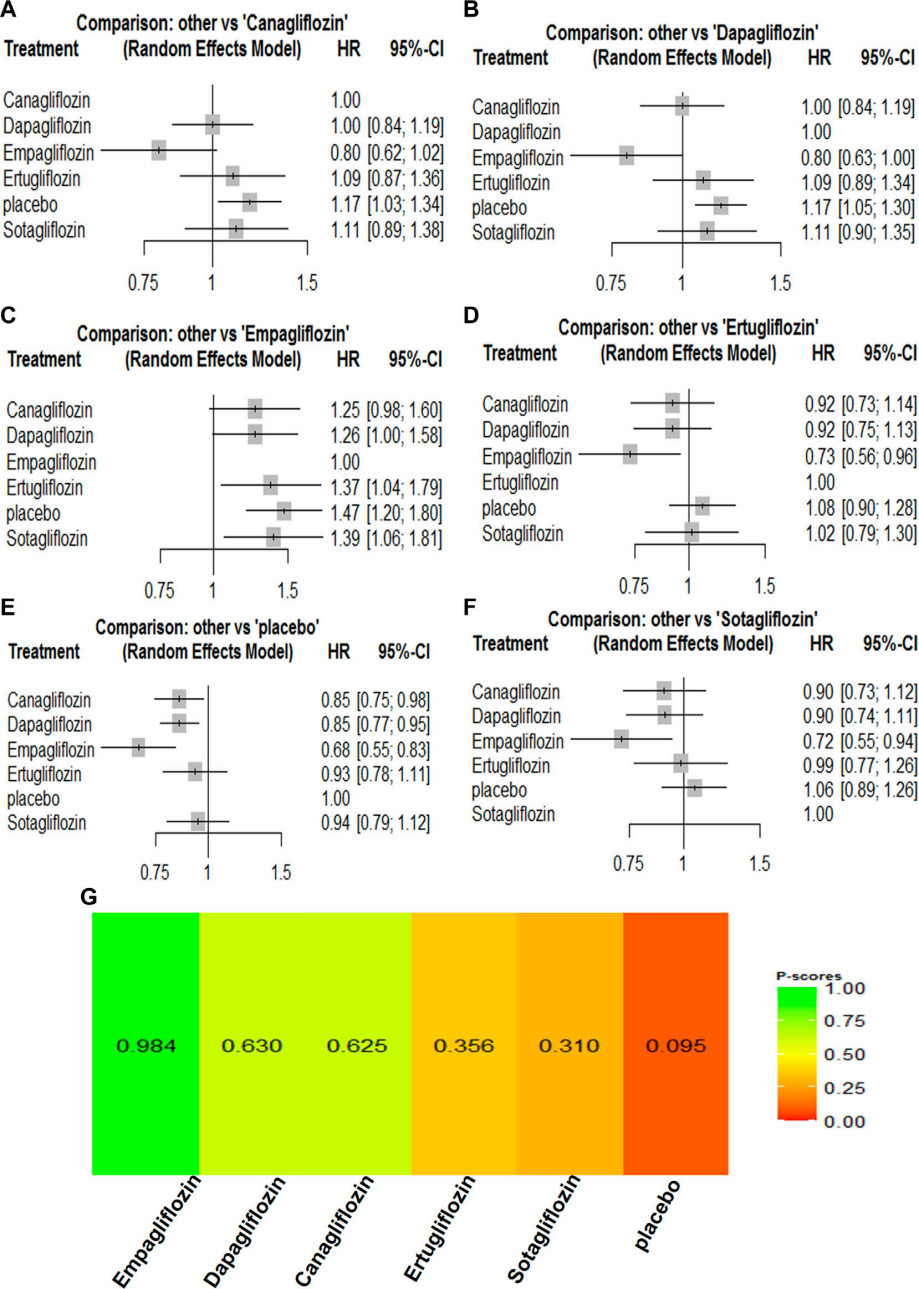
Forest Plots and Ranking Plots of Network Meta-analysis of all trials for All-cause Mortality. **(A)** Forest Plots of other drugs compare to canagliflozin. **(B)** Forest Plots of other drugs compare to dapagliflozin. **(C)** Forest Plots of other drugs compare to empagliflozin. **(D)** Forest Plots of other drugs compare to ertugliflozin. **(E)** Forest Plots of other drugs compare to placebo. **(F)** Forest Plots of other drugs compare to sotagliflozin. All outcomes are expressed as hazard ratios (HRs) for treatment vs the comparator and 95% credible intervals (95%-CI). For example, the HRs in all-cause mortality for dapagliflozin compared to canagliflozin is 1.00 (95%-CI 0.84 to 1.19). The x-axis scale indicates the range of the HRs. **(G)** Ranking Plots of Network Meta-analysis. Plots below the forest plots show for the rank of each drug class and ranking descending from left to right, The p-score represents the power of the ranking.

All the included trials reported all-cause mortality for the 67,823 participants. There were 4,824 events (7.11%) among the 67,823 participants. Compared with the placebo groups, canagliflozin [HR, 0.85 (95%CI, 0.75–0.98)], dapagliflozin [HR, 0.85 (95%CI, 0.77–0.95)], empagliflozin [HR, 0.68 (95%CI, 0.55–0.83)], ertugliflozin [HR, 0.93 (95%CI, 0.78–1.11)], and sotagliflozin [HR, 0.94 (95%CI, 0.79–1.12)] were associated with reductions in all-cause mortality (Supplementary Table S2; and Figure 2). The P-score rank showed the drug rankings for reducing all-cause mortality, which were as follows: empagliflozin > dapagliflozin > canagliflozin > ertugliflozin > sotagliflozin (Figure 2).

### 3.3 Network meta-analyses of SGLT2 inhibitors on cardiovascular outcomes

**FIGURE 3:**
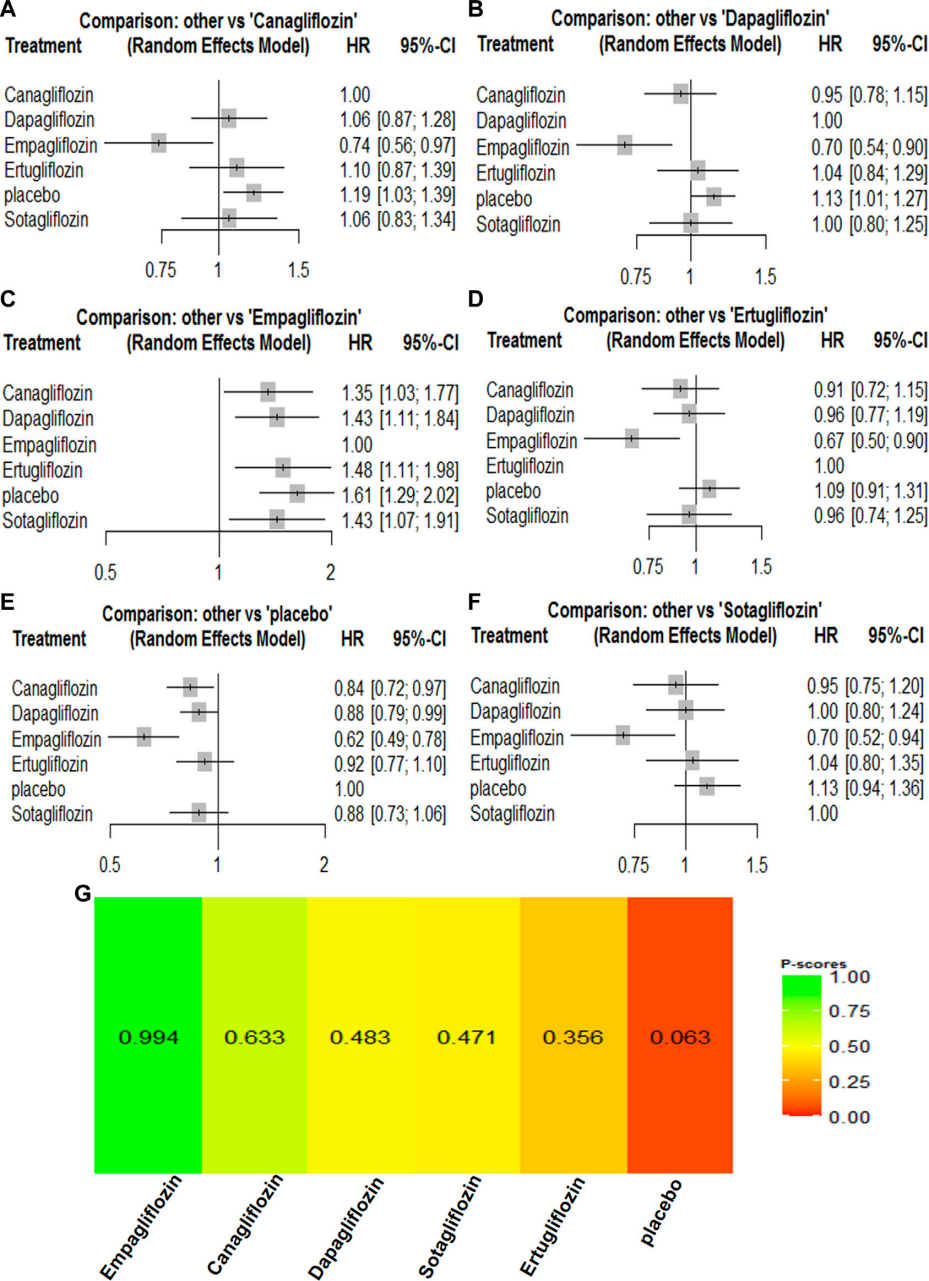
Forest Plots and Ranking Plots of Network Meta-analysis of all trials for Cardiovascular Mortality. **(A)** Forest Plots of other drugs compare to placebo. **(B)** Forest Plots of other drugs compare to dapagliflozin. **(C)** Forest Plots of other drugs compare to empagliflozin. **(D)** Forest Plots of other drugs compare to ertugliflozin. **(E)** Forest Plots of other drugs compare to canagliflozin. **(F)** Forest Plots of other drugs compare to sotagliflozin. All effect estimates are expressed as hazard ratios (HRs) for treatment vs the comparator and 95% credible intervals (95%-CI). For example, the HRs in cardiovascular mortality for dapagliflozin compared to canagliflozin is 1.06 (95%-CI 0.87 to 1.28). The x-axis scale indicates the range of the HRs. **(G)** Ranking Plots of Network Meta-analysis. Plots below the forest plots show for the rank of each drug class and ranking descending from left to right, The p-score represents the power of the ranking.

**FIGURE 4:**
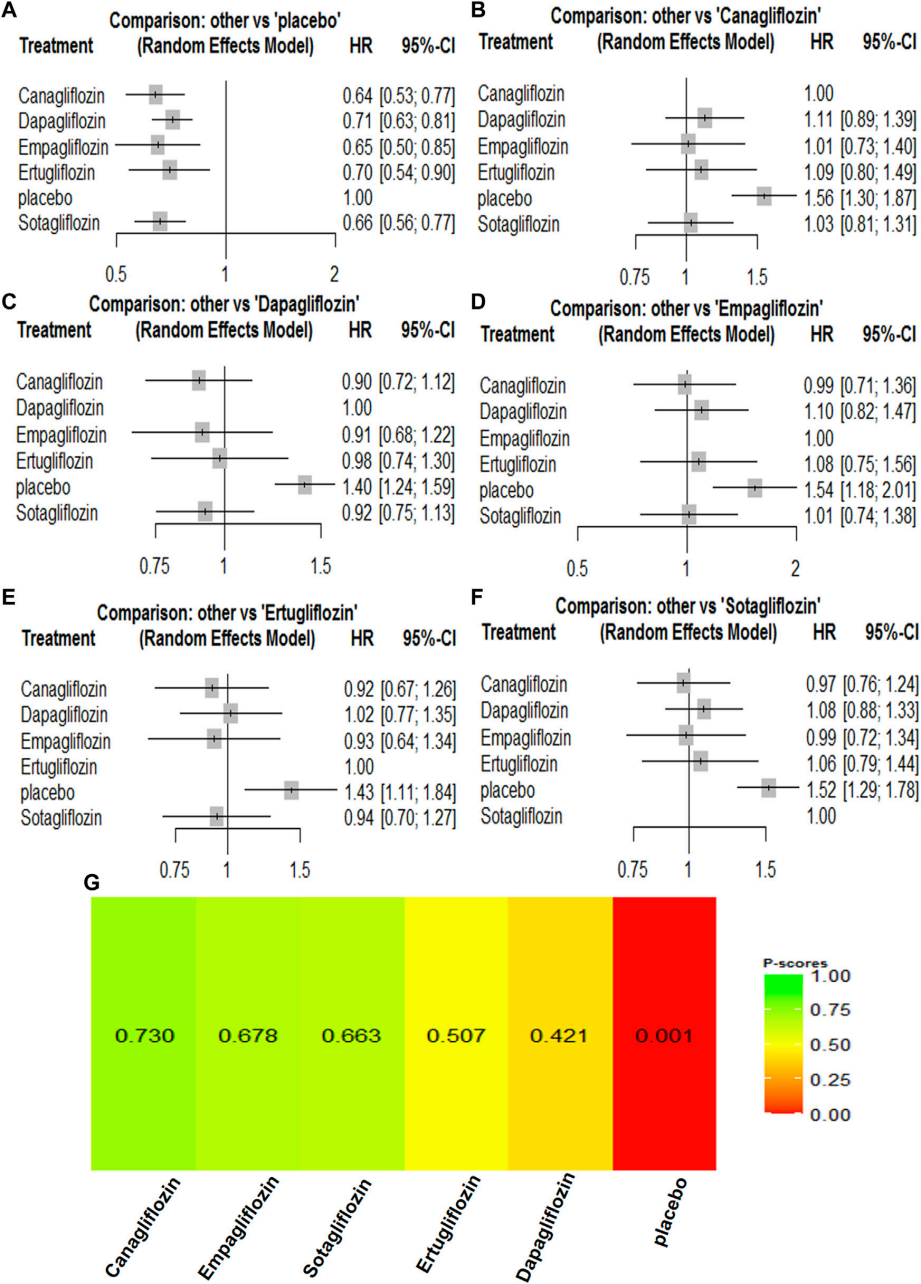
Forest Plots and Ranking Plots of Network Meta-analysis of all trials for Hospitalization for Heart Failure. **(A)** Forest Plots of other drugs compare to canagliflozin. **(B)** Forest Plots of other drugs compare to dapagliflozin. **(C)** Forest Plots of other drugs compare to empagliflozin. **(D)** Forest Plots of other drugs compare to ertugliflozin. **(E)** Forest Plots of other drugs compare to placebo. **(F)** Forest Plots of other drugs compare to sotagliflozin. All effect estimates are expressed as hazard ratios (HRs) for treatment vs the comparator and 95% credible intervals (95%-CI). For example, the HRs in hospitalization for heart failure for canagliflozin compared to placebo is 0.64 (95%-CI 0.53 to 0.77). The x-axis scale indicates the range of the HRs. **(G)** Ranking Plots of Network Meta-analysis. Plots below the forest plots show for the rank of each drug class and ranking descending from left to right, The p-score represents the power of the ranking.

For cardiovascular outcomes, nine trials that had enrolled 67,823 participants reported cardiovascular mortality and hospitalization for heart failure. All SGLT2 inhibitors lowered the risk of all-cause mortality and hospitalization for heart failure when compared with placebo (Supplementary Table S2). Empagliflozin [HR, 0.74 (95%CI, 0.56–0.97)] was associated with a reduction in cardiovascular mortality compared with canagliflozin. Canagliflozin [HR, 0.95 (95%CI, 0.78–1.15)] was associated with a reduction in cardiovascular mortality compared with dapagliflozin, while dapagliflozin [HR, 1.00 (95%CI, 0.80–1.24)] was associated with a reduction in cardiovascular mortality compared with sotagliflozin. Compared with ertugliflozin, sotagliflozin [HR, 0.96 (95%CI, 0.74–1.25)] reduced the risk of a cardiovascular mortality event. The cardiovascular mortality rankings were as follows: empagliflozin > canagliflozin > dapagliflozin > sotagliflozin > ertugliflozin (Supplementary Table S2; Figure 3). Canagliflozin [HR, 0.99 (95%CI, 0.71–1.36)] was associated with fewer hospitalizations compared with empagliflozin. Empagliflozin [HR, 0.99 (95%CI, 0.72–1.34)] was associated with a reduction in hospitalization for heart failure compared with sotagliflozin, while sotagliflozin [HR, 0.94 (95%CI, 0.70–1.27)] was associated with a reduction in hospitalization for heart failure compared with ertugliflozin. Ertugliflozin [HR, 0.98 (95%CI, 0.74–1.30)] was associated with a reduction in hospitalization for heart failure compared with dapagliflozin. The rankings for reducing hospitalization for heart failure were as follows: canagliflozin > empagliflozin > sotagliflozin > ertugliflozin > dapagliflozin (Supplementary Table S2; Figure 4). Canagliflozin and empagliflozin seemed to have the same efficacy in reducing hospitalization for heart failure, but empagliflozin had advantage in reducing cardiovascular mortality.

### 3.4 Network meta-analyses of SGLT2 inhibitors on composite renal outcome

**FIGURE 5:**
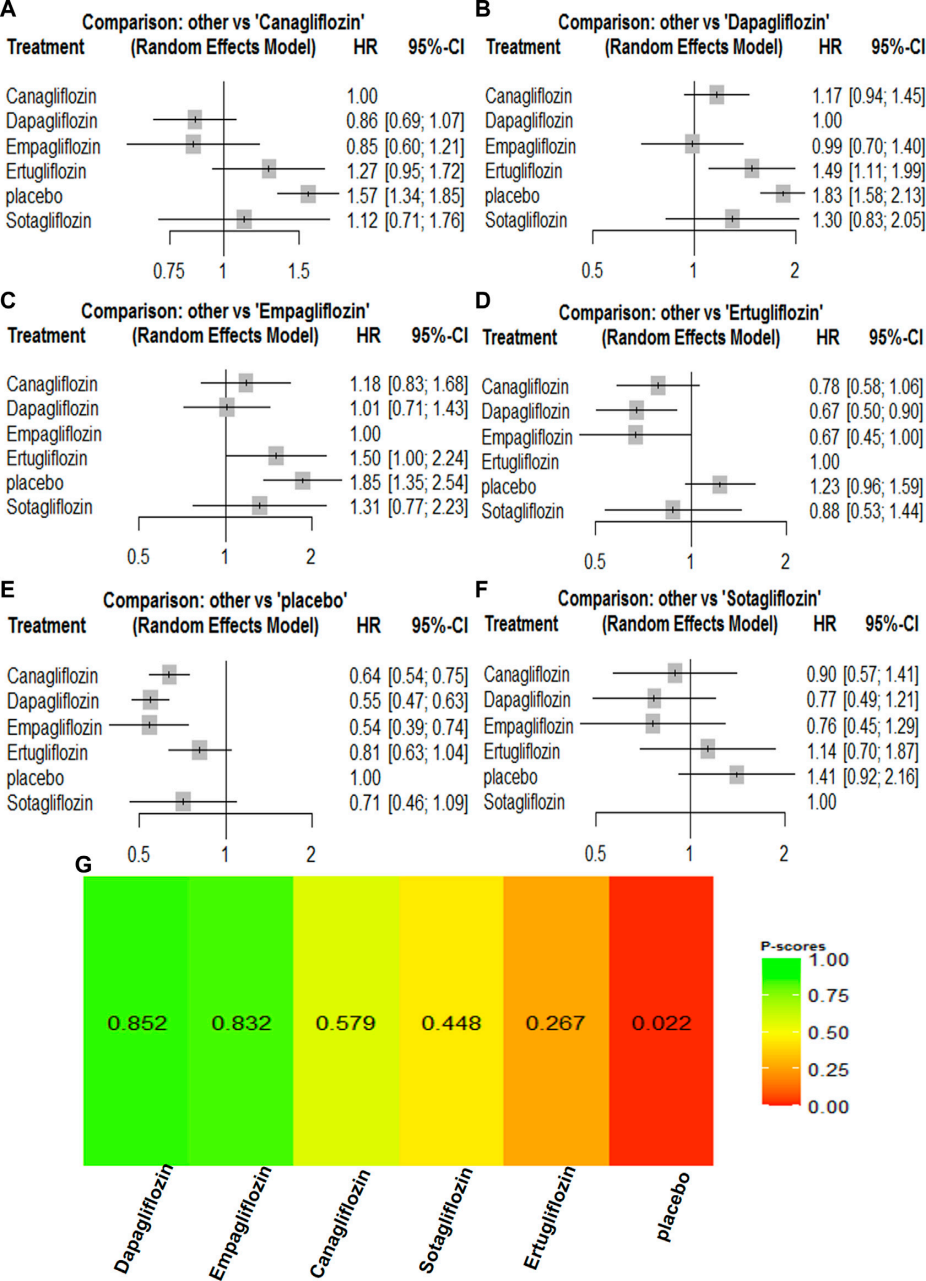
Forest Plots and Ranking Plots of Network Meta-analysis of all trials for Composite Renal Outcome. **(A)** Forest Plots of other drugs compare to canagliflozin. **(B)** Forest Plots of other drugs compare to dapagliflozin. **(C)** Forest Plots of other drugs compare to empagliflozin. **(D)** Forest Plots of other drugs compare to ertugliflozin. **(E)** Forest Plots of other drugs compare to placebo. **(F)** Forest Plots of other drugs compare to sotagliflozin. All effect estimates are expressed as hazard ratios (HRs) for treatment vs the comparator and 95% credible intervals (95%-CI). For example, the HRs in composite renal outcome for dapagliflozin compared to canagliflozin is 0.86 (95%-CI 0.69 to 1.07). The x-axis scale indicates the range of the HRs. **(G)** Ranking Plots of Network Meta-analysis. Plots below the forest plots show for the rank of each drug class and ranking descending from left to right, The p-score represents the power of the ranking.

A composite renal outcome consisted of new or worsening persistent microalbuminuria or macroalbuminuria, new or worsening chronic kidney disease, doubling of serum creatinine, end-stage renal disease, renal transplant, or renal death. For the composite renal outcomes, nine trials enrolling 63,079 patients were included. Compared with the control groups, canagliflozin [HR, 0.64 (95%CI, 0.54–0.75)], dapagliflozin [HR, 0.55 (95%CI, 0.47–0.63)], empagliflozin [HR, 0.54 (95%CI, 0.39–0.74)], ertugliflozin [HR, 0.70 (95%CI, 0.54–0.90)], and sotagliflozin [HR, 0.66 (95%CI, 0.56–0.77)] were associated with a reduction in the renal composite outcome. Dapagliflozin [HR, 1.01 (95%CI, 0.71–1.43)] was associated with a reduction in the renal composite compared with empagliflozin, while empagliflozin [HR, 0.85 (95%CI, 0.60–1.21)] was associated with a reduction in the renal composite compared with canagliflozin. Canagliflozin [HR, 0.90 (95%CI, 0.57–1.41)] was associated with a reduction in the renal composite compared with sotagliflozin, while sotagliflozin [HR, 0.88 (95%CI, 0.53–1.44)] was associated with a reduction in the renal composite compared with ertugliflozin. Dapagliflozin was most likely to have the best results for renal composite outcomes, and the rankings for the renal composite outcome results were as follows: dapagliflozin > empagliflozin > canagliflozin > sotagliflozin > ertugliflozin (Supplementary Table S2; Figure 5).

## 4 Discussion

This network meta-analysis was the first to incorporate the large and recently published randomized controlled trial about SGLT2 inhibitors in the management of T2DM. SGLT2 inhibitors are the latest recommended first-line hypoglycemic drugs, and our study including dapagliflozin, canagliflozin, tofogliflozin, luseogliflozin, ipragliflozin, empagliflozin, ertugliflozin, and sotagliflozin to provide evidence to help select the most appropriate SGLT2 inhibitor for patients with different cardiovascular and renal complication risk factors. The results of our network meta-analysis suggested the following: 1) For cardiovascular outcomes, all SGLT2 inhibitors reduced the risk of all-cause mortality and hospitalization for heart failure compared with placebo, and the rankings for cardiovascular mortality were empagliflozin > canagliflozin > dapagliflozin > sotagliflozin > ertugliflozin, while the rankings for reduced hospitalization for heart failure were canagliflozin > empagliflozin > sotagliflozin > ertugliflozin > dapagliflozin. 2) For a renal composite outcome, dapagliflozin was the most likely to show the best renal composite outcomes compared with the control group. The rankings were dapagliflozin > empagliflozin > canagliflozin > sotagliflozin > ertugliflozin.

T2DM is a systemic metabolic disease that affects the microvascular and macrovascular systems. Hyperglycemia is the primary risk factor for microvascular complications, such as nephropathy, retinopathy, and neuropathy ((UKPDS) Group, 1998; Diabetes Control and Complications Trial Research Group, 1994). Meaningful benefits of improved blood glucose on macrovascular complications were more pronounced after 10 years or more (Holman et al., 2008). SGLT2 inhibitors may be vital blood glucose lowering agents for T2DM treatment because they exert multiple beneficial metabolic effects such as controlling body weight, uric acid levels, and blood pressure (BP) (Toyama et al., 2019). For cardiovascular events, the precise mechanism of the SGLT2 inhibitor-induced cardiovascular benefit remains to be determined, but previous studies have shown that SGLT2 inhibitors may prevent cardiovascular outcomes by regulating dyslipidemia, restoring normal endothelial function, inhibiting cardiac remodeling, and inhibiting the evolution of monocyte–macrophage foam cells (Terasaki et al., 2015; Kang et al., 2020; Lee et al., 2020; Park et al., 2021). Previous reports suggested that SGLT2 inhibitors may also lead to vasodilation and positive inotropic effects at the angiotensin type II receptor during simultaneous blockade of the renin–angiotensin–aldosterone system (the RAAS hypothesis) (Filippatos et al., 2019). All the above are possible mechanisms of the cardioprotective effect of SGLT2 inhibitors. In addition, Previous meta-analysis showed a moderate benefit of SGLT2 inhibitors for major adverse atherosclerotic cardiovascular events and a significant benefit in reducing hospitalizations for heart failure and renal disease progression, regardless of the presence of atherosclerotic cardiovascular disease or a history of heart failure, but there are differences in the dose, efficacy, and safety among different SGLT2 inhibitors (Zelniker et al., 2019). In 2015, Empagliflozin as a SGLT2 inhibitor first published data with (Cardio vascular) CV outcomes. Compared with other SGLT2 inhibitors, empagliflozin has the highest selectivity for SGLT2. Although SGLT2 expression in the myocardium is negligible, SGLT2 inhibitors can directly reduce Na + -H+ exchanger (NHE) activity in cardiomyocytes through the binding site of SGLT2 on NHE. It is well known that failing cardiomyocytes display enhanced intracellular sodium levels, at least in part on account of increased activity of the sarcolemmal NHE, resulting in associated Ca^2+^ efflux from mitochondria leading to exacerbation of cell function and reduced antioxidant capacity (Darmellah et al., 2007; Bell and Yellon, 2018; Bertero et al., 2018). Thus, SGLT2 inhibitors can reduce the incidence of ventricular arrhythmias and sudden cardiac death by reducing cardiomyocyte NHE activity leading to a reduction in intracellular sodium and restoration of mitochondrial calcium handling, as well as sudden cardia death. The hypothesis may the advantage effects of empagliflozin on cardiovascular mortality. Previous studies have also found that Empagliflozin has a direct pleiotropic effect on isolated failing human myocardium, as well as on the diastolic function of healthy and diseased mouse myocardium, which has direct pleiotropic effects on the myocardium by improving myocardial diastolic stiffness and diastolic function, and these effects were not related to diabetes (Pabel et al., 2018). Empagliflozin switches myocardial fuel utilization from the low-yield energy-producing glucose metabolism to (ketone bodies) KB (free fatty acid) FFA, and (branched -chain amino acid) BCAA, thereby ameliorating myocardial energy, boosting (left ventricular) LV systolic function, as well as improving adverse LV remodeling (Santos-Gallego et al., 2019). Moreover, canagliflozin seemed to have the same efficacy in reducing hospitalization for heart failure as empagliflozin. Canagliflozin is also a low-potency sodium–glucose co-transporter type 1 (SGLT1) inhibitor, which may distinguish it from other SGLT2 inhibitors (Scheen, 2015). The SGLT1 transporters are more widely distributed in heart. SGLT1 is the major transporter for intestinal glucose absorption, and intestinal inhibition of SGLT1 results in glucose–galactose malabsorption, which may also show better hypoglycemic results with canagliflozin. Another meta-analysis also indicated that canagliflozin, which is a SGLT2 inhibitor only, decreases systolic blood pressure in a dose-dependent manner (0.87 mmHg for every additional 100 mg of canagliflozin), which may explain its additional cardiovascular benefits (Baker et al., 2014).

In terms of renal events, previous studies have not fully revealed the mechanism by which SGLT2 inhibitors benefit renal outcomes in individuals with T2DM. The nephroprotective effect of SGLT2 inhibitors in patients with T2DM may involve multiple mechanisms, which mainly include the following aspects: 1) SGLT2 inhibitors inhibit proximal tubular sodium reabsorption, which results in increased sodium delivery to the juxtaglomerular apparatus, decreased intraglomerular pressure, and normalized ultrafiltration, which decreases the kidney’s injury and reduces albuminuria (Vallon and Thomson, 2017); 2) reduced activation of the intrarenal renin–angiotensin–aldosterone system, which lowers blood pressure and helps to reduce glomerular hyperfiltration (Ravindran and Munusamy, 2022). Some reports speculate that SGLT2 inhibitors show similar effects as that of angiotensin blockade, leading to a decrease in the initiation and functional decline in the estimated glomerular filtration rate (eGFR), which may contribute to long-term preservation of renal function (Nespoux and Vallon, 2018); 3) increased ketone bodies in individuals with type 2 diabetes. Similar to fatty acids, ketone bodies could be used as an alternative fuel for mitochondria to synthesize ATP. Moreover, erythropoietin levels were also increased, which could improve renal tissue oxygenation, transfer fuel selection from glucose to ketone bodies, help to improve mitochondrial function, and help to attenuate inflammation (Ferrannini, 2017); and 4) protect against hypoxia and oxidative stress, regulate autophagy, and improve fibrosis. The results of our analysis indicated that dapagliflozin has the best effect on renal outcomes in patients with T2DM. However, there has been no report on the possible mechanism by which dapagliflozin has better renal events than other SGLT2 inhibitors.

A main limitation of this network analysis is the heterogeneity of the included trials. The sources of this heterogeneity mainly include differences in clinical settings as well as differences in national and ethnic groups, although the consistency of the findings alleviates this concern. Moreover, in individual trials, patients included differences in baseline renal function levels, which may affect renal outcomes. The definition of the cardiovascular risk was inconsistent across the trials that were included in the network analysis of patients with an increased cardiovascular risk.

## 5 Conclusion

In conclusion, our network analysis incorporating the latest and most comprehensive RCTs for SGLT2 inhibitors established a solid evidence base that verified a prominent effect of dapagliflozin and canagliflozin on renal and cardiovascular outcomes compared with similar SGLT2 inhibitors. These class agents also reduced all-cause mortality and showed benefits regardless of the patient’s sex or if they had diabetes.

## Data Availability

The original contributions presented in the study are included in the article/Supplementary Material, further inquiries can be directed to the corresponding authors.

## Abbreviations

T2DM, Type 2 diabetes mellitus; SGLT-2, Sodium-glucose cotransporter-2; DKD, diabetic kidney disease; ESRD, End-stage renal disease; RCT, Randomized controlled trial; PRISMA, Preferred reporting items for systematic reviews and meta-analyses; FDA, Food and Drug Administration; MACE, Major adverse cardiovascular events; HR, Hazard ratios; CI, Confidence interval; eGFR, Estimated glomerular filtration rate; BP, Blood pressure; CVOT, Cardiovascular Outcomes Trials; NHE, Na + -H+ exchanger; KB, Ketone bodies; FFA, Free fatty acid; BCAA, branched-chain amino acid; LV, left ventricular; CV, Cardio vascular; SGLT-1, sodium–glucose co-transporter type 1 inhibitor; HIF1, hypoxia-inducible factor 1.
