# Supplementary material for: Cardiovascular and renal outcomes with sodium glucose co-transporter 2 inhibitors in patients with type 2 diabetes mellitus: A system review and network meta-analysis
Source: Front Pharmacol. 2022 Nov 24;13:986186. doi: 10.3389/fphar.2022.986186 (PMC9731650; doi:10.3389/fphar.2022.986186)
Supplement: Supplementary file 1 [file DataSheet1.PDF]

Table1. Study Participant Characteristics

	group	a. Participant population	b. Mean age	c. Mean duration of diabetes	d. Mean follow up（Months）	e. First author
**CANVAS**	/	Type 2 Diabetes	62.4(8.0)	13.4(7.5)	12	Neal, B.
**CANVAS-R**	/	Type 2 Diabetes	64.0(8.4)	13.7(7.9)	12	Neal, B.
**EMPA-REG OUTCOME**	Empagliflozin	Type 2 Diabetes	/	/	48	Bernard Zinman
	Placebo		/	/	48	
**CREDENCE**	Canagliflozin	Diabetes with CKD	62.9±9.2	15.8±8.6	42	V. Perkovic
	Placebo		63.0±9.2	15.5±8.7	42	
**DECLARE–TIMI 58**	Dapagliflozin	Type 2 Diabetes	63.9±6.8	11.0(6.0-16.0)	48	Wiviott, S. D.
	Placebo		64.0±6.8	10.0(6.0-16.0)	48	
**VERTIS CV**	Ertugliflozin	Type 2 Diabetes	64.4±8.1	12.9±8.3	60	C.P. Cannon
	Placebo		64.4±8.0	13.1±8.4	60	
**SOLOIST-WHF**	Sotagliflozin	Diabetes and Recent Worsening Heart Failure	69(63-76)	/	18	D.L. Bhatt
	Placebo		70(64-76)	/	18	
**DAPA-CKD**	Dapagliflozin	Diabetes with CKD	61.8±12.1	/	32	Hiddo J.L
	Placebo		61.9±12.1	/	32	
**DAPA-HF**	Dapagliflozin	no diabetic with Heart Failure and Reduced Ejection Fraction	66.2±11.0	/	24	J.J.V. McMurray
	Placebo		66.5±10.8	/	24	
**SCORED**	Sotagliflozin	Diabetes with CKD	69 (63–74)	/	24	Deepak L. Bhatt
	Placebo		69 (63–74)	/	24	

The Table represents data from studies stratified by the intervention and comparator. Control refers to placebo.

Table 2. Pairwise comparisons of the efficacy and safety of the six drugs included in the study, reported in alphabetical order.

All-cause mortality					
Canagliflozin	1.00(0.84-1.19)	1.25(0.98-1.60)	0.92(0.73-1.14)	0.85(0.75-0.98)	0.90(0.73-1.12)
1.00(0.84-1.19)	Dapagliflozin	1.26(1.00-1.58)	0.92(0.75-1.13)	0.85(0.77-0.95)	0.90(0.74-1.11)
0.80(0.62-1.02)	0.80(0.63-1.00)	Empagliflozin	0.73(0.56-0.96)	0.68(0.55-0.83)	0.72(0.55-0.94)
1.09(0.87-1.36)	1.09(0.89-1.34)	1.37(1.04-1.79)	Ertugliflozin	0.93(0.78-1.11)	0.99(0.77-1.26)
1.17(1.03-1.34)	1.17(1.05-1.30)	1.47(1.20-1.80)	1.08(0.90-1.28)	Placebo	1.06(0.89-1.26)
1.11(0.89-1.38)	1.11(0.90-1.35)	1.39(1.06-1.81)	1.02(0.79-1.30)	0.94(0.79-1.12)	Sotagliflozin
Cardiovascular mortality					
Canagliflozin	0.95(0.78-1.15)	1.35(1.03-1.77)	0.91(0.72-1.15)	0.84(0.72-0.97)	0.95(0.75-1.20)
1.06(0.87-1.28)	Dapagliflozin	1.43(1.11-1.84)	0.96(0.77-1.19)	0.88(0.79-0.99)	1.00(0.80-1.24)
0.74(0.56-0.97)	0.70(0.54-0.90)	Empagliflozin	0.67(0.50-0.90)	0.62(0.49-0.78)	0.70(0.52-0.94)
1.10(0.87-1.39)	1.04(0.84-1.29)	1.48(1.11-1.98)	Ertugliflozin	0.92(0.77-1.10)	1.04(0.80-1.35)
1.19(1.03-1.39)	1.13(1.01-1.27)	1.61(1.29-2.02)	1.09(0.91-1.31)	Placebo	1.13(0.94-1.36)
1.06(0.83-1.34)	1.00(0.80-1.25)	1.43(1.07-1.91)	0.96(0.74-1.25)	0.88(0.73-1.06)	Sotagliflozin

Renal composite					
Canagliflozin	1.17 (0.94-1.45)	1.18(0.83-1.68)	0.78(0.58-1.06)	0.64(0.54-0.75)	0.90(0.57-1.41)
0.86(0.69-1.07)	Dapagliflozin	1.01 (0.71-1.43)	0.67 (0.50-0.90)	0.55 (0.47-0.63)	0.77 (0.49-1.21)
0.85(0.60-1.21)	0.99(0.70-1.40)	Empagliflozin	0.67 (0.45-1.00)	0.54(0.39-0.74)	0.76(0.45-1.29)
1.27 (0.95-1.72)	1.49(1.11-1.99)	1.50(1.00-2.24)	Ertugliflozin	0.81(0.63-1.04)	1.14(0.70-1.87)
1.57(1.34-1.85)	1.83(1.58-2.13)	1.85(1.35-2.54)	1.23 (0.96-1.59)	Placebo	1.41(0.92-2.16)
1.12(0.71-1.76)	1.30 (0.83-2.05)	1.31(0.77-2.23)	0.88(0.53-1.44)	0.71(0.46-1.09)	Sotagliflozin
Hospitalization for heart failure					
Canagliflozin	0.90(0.72-1.12)	0.99(0.71-1.36)	0.92 (0.67-1.26)	0.64 (0.53-0.77)	0.97 (0.76-1.24)
1.11 (0.89-1.39)	Dapagliflozin	1.10(0.82-1.47)	1.02 (0.77-1.35)	0.71 (0.63-0.81)	1.08(0.88-1.33)
1.01(0.73-1.40)	0.91(0.68-1.22)	Empagliflozin	0.93 (0.64-1.34)	0.65(0.50-0.85)	0.99 (0.72-1.34)
1.09(0.80-1.49)	0.98(0.74-1.30)	1.08(0.75-1.56)	Ertugliflozin	0.70(0.54-0.90)	1.06(0.79-1.44)
1.56(1.30-1.87)	1.40(1.24-1.59)	1.54(1.18-2.01)	1.43(1.11-1.84)	Placebo	1.52(1.29-1.78)
1.03(0.81-1.31)	0.92(0.75-1.13)	1.01 (0.74-1.38)	0.94(0.70-1.27)	0.66(0.56-0.77)	Sotagliflozin

The data in each grid represent the HRs, and 95 % CIs. Column-defining drugs are compared to the row-defining drugs.
